# Long term survival of cirrhotics following admission to a uk intensive care unit - a subgroup of patients with ´resilient´ cirrhosis

**DOI:** 10.1186/2197-425X-3-S1-A689

**Published:** 2015-10-01

**Authors:** J Lloyd-Evans, T Pembroke, A Godkin

**Affiliations:** Department of Anaesthetics and Intensive Care, University Hospital of Wales, Cardiff, United Kingdom; Department of Gastroenterology and Hepatology, University Hospital of Wales, Cardiff, United Kingdom; Infection and Immunology, School of Medicine, Cardiff University, Cardiff, United Kingdom

## Introduction

Patients with cirrhosis who are admitted to the ICU are considered to have a poor prognosis. Previous studies have sought to identify markers of high predictive value in patients who will not survive their critical care stay including the need for mechanical ventilation, vasopressor support, GI bleeding and APACHE III score. These studies have focused upon early prognosis, rather than long term survival.

## Objectives

We sought to validate previously described outcome predictors and review long-term survival in a cohort of cirrhotic patients admitted to the ICU.

## Methods

We performed a retrospective analysis of cirrhotic patients admitted to adult ICU, University Hospital of Wales (Cardiff, UK) between 2009-14. Admission information was collated from the Intensive Care National Audit and Research Database. Child-Pugh, Model for End-stage Liver Disease-Na (MELD-Na) and Acute Physiology and Chronic Health Evaluation (APACHE II) scores were derived from data on admission to critical care. Statistical analyses were conducted with SPSS.

## Results

One hundred and fifty one cirrhotics (2.8% of all critical care admissions) were admited to the ICU with a mean age of 54 (range 22-80) and a male preponderance (65.6%). The predominant aetiology was alcoholic liver disease (ALD, 107/151, 70.9%). The reason for ICU admission were variceal bleed (29.8%); sepsis (25.2%); decompensation (13.9%); non variceal bleed (5.3%) and other (26%). ICU mortality was 43.7% for the 151 patients. Mean follow up after ICU discharge was 788 days (range 26-1722).

Hyponatraemia (p < 0.001), coagulopathy (p = 0.02), APACHE II (p = 0.004) and MELD-Na scores (p < 0.001) were significantly associated with mortality. Patients diagnosed as decompensation with no obvious underlying precipitant had a worse prognosis than those with precipitants (e.g. variceal bleeding, sepsis) which was maintained at follow up (p = 0.05). Child´s A, B and C stage patients had a mean overall survival of 1274, 976 and 690 days respectively (p = 0.01).

Seventy eight patients were discharged from hospital. Only 6 of these died, all with Child´s B liver disease. Only attending outpatient follow up clinic was associated with increased survival (p = 0.01, 1373 v 1668 days).

## Conclusions

Nearly half of all cirrhotics admitted to the ICU die before discharge, and two thirds of all patients have alcohol as an aetiological factor, emphasizing the burden on our society. As expected, commonly used scoring systems predict short-term outcome, but often do not reflect long-term survival.

Cirrhotics who survive the challenge of ICU admission seem to have quite marked physiological resilence and capacity for recovery with a good long term prognosis. Interestingly only patients with Child´s B cirrhosis continued to demonstrate a post hospital discharge mortality, warranting further investigation for underlying reasons.

